# Development of a lateral flow dipstick test for the detection of 4 strains of *Salmonella* spp. in animal products and animal production environmental samples based on loop-mediated isothermal amplification

**DOI:** 10.5713/ab.22.0151

**Published:** 2022-09-07

**Authors:** Wirawan Nuchchanart, Prapasiri Pikoolkhao, Chalermkiat Saengthongpinit

**Affiliations:** 1 Department of Animal Science, Faculty of Agriculture at Kamphaeng Saen, Kasetsart University, Nakhon Pathom 73140, Thailand; 2 Center for Agricultural Biotechnology, Kasetsart University, Kamphaeng Saen Campus, Nakhon Pathom 73140, Thailand; 3 Center of Excellence on Agricultural Biotechnology: (AG-BIO/MHESI), Bangkok 10900, Thailand; 4 Department of Veterinary Public Health, Faculty of Veterinary Medicine, Kasetsart University, Nakhon Pathom 73140, Thailand

**Keywords:** Animal Products, Detection, *InvA* Gene, Lateral Flow Dipstick (LFD), Loop-mediated Isothermal Amplification, *Salmonella* spp

## Abstract

**Objective:**

This study aimed to develop loop-mediated isothermal amplification (LAMP) combined with lateral flow dipstick (LFD) and compare it with LAMP-AGE, polymerase chain reaction (PCR), and standard *Salmonella* culture as reference methods for detecting *Salmonella* contamination in animal products and animal production environmental samples.

**Methods:**

The SalInvA01 primer, derived from the *InvA* gene and designed as a new probe for LFD detection, was used in developing this study. Adjusting for optimal conditions by temperature, time, and reagent concentration includes evaluating the specificity and limit of detection. The sampling of 120 animal product samples and 350 animal production environmental samples was determined by LAMP-LFD, comparing LAMP-AGE, PCR, and the culture method.

**Results:**

*Salmonella* was amplified using optimal conditions for the LAMP reaction and a DNA probe for LFD at 63°C for 60 minutes. The specificity test revealed no cross-reactivity with other microorganisms. The limit of detection of LAMP-LFD in pure culture was 3×10^2^ CFU/mL (6 CFU/reaction) and 9.01 pg/μL in genomic DNA. The limit of detection of the LAMP-LFD using artificially inoculated in minced chicken samples with 5 hours of pre-enrichment was 3.4×10^4^ CFU/mL (680 CFU/reaction). For 120 animal product samples, *Salmonella* was detected by the culture method, LAMP-LFD, LAMP-AGE, and PCR in 10/120 (8.3%). In three hundred fifty animal production environmental samples, *Salmonella* was detected in 91/350 (26%) by the culture method, equivalent to the detection rates of LAMP-LFD and LAMP-AGE, while PCR achieved 86/350 (24.6%). When comparing sensitivity, specificity, positive predictive value, and accuracy, LAMP-LFD showed the best results at 100%, 95.7%, 86.3%, and 96.6%, respectively. For Kappa index of LAMP-LFD, indicated nearly perfect agreement with culture method.

**Conclusion:**

The LAMP-LFD *Salmonella* detection, which used *InvA* gene, was highly specific, sensitive, and convenient for identifying *Salmonella*. Furthermore, this method could be used for *Salmonella* monitoring and primary screening in animal products and animal production environmental samples.

## INTRODUCTION

Foodborne pathogens cause a wide range of diseases with major consequences for human health and the economy. A foodborne disease outbreak occurs when two or more cases of a similar illness occur as a result of the consumption common food. The World Health Organization reported that, poisoned foods and water affect over 600 million people globally, resulting 4.2 million deaths per year [1]. *Salmonella* is regarded as a serious public health problem, closely associated with food-borne illness outbreaks all around the world [2]. According to the Centers for Disease Control and Prevention (CDC) reported the *Salmonella* causes 1.35 million illnesses, 2.65 thousand hospitalizations with confirmed infections and 420 deaths occur in the United States each year [3]. Salmonellosis is commonly spread by consuming food or water contaminated with sick animal feces. The infection can be transmitted by direct contact with infected animals or through the eating of animal-derived foods such as chicken, swine, fish, shellfish, and animal products [4]. In order to maintain a safe food supply and lower *Salmonella* infection levels in the future, it is necessary to monitor and test for the appearance of *Salmonella* [5].

The standard *Salmonella* culture method for the detection and identification of *Salmonella* in food samples relies upon bacteria growing in culture media such as pre-enrichment, selective enrichment, selective plating, biochemical testing, and serological testing. These procedures are reliable, simple, and inexpensive, but they take a few days [6]. Molecular technology was developed to investigate food-borne pathogens such as *Salmonella* in a variety of foods, especially with the advent and ongoing development of molecular diagnostic tools [7]. The first commercially available polymerase chain reaction (PCR)-based molecular food detection technology was released in 1992. Other methods, including real-time PCR and multiplexing, became accessible in the years following.

Since 2000, a new generation of amplification techniques based on loop-mediated isothermal amplification, or LAMP, has been created by Japanese researcher Prof. Tsugunori Notomi and colleagues as a popular tool for pathogen inquiry [8]. LAMP is based on the activity of the *Bst* DNA polymerase enzyme, which may produce a particular strand of nucleotide and 4 to 6 strands of primers that can bind to specific target genes at up to 6 sites at 60°C to 65°C. As a result, a highly specialized LAMP product was created. LAMP is becoming increasingly popular since it does not require expensive equipment such as a thermal cycler or PCR machine [9]. LAMP products can be determined using a variety of techniques, involving turbidimeters, fluorescent agents, gel electrophoresis, and the lateral flow dipstick (LFD) immune-chromatographic assay [10]. The LFD test is a simple diagnostic method for confirming whether a target sample exists or not, such as diseases or biomarkers in humans or animals, or contaminants in water, food, or animal feeds. The most well-known type of lateral flow rapid test strip is the pregnancy test [11]. The LFD assay is able to detect DIG-conjugated LAMP products that were formerly hybridized with a DNA probe labeled with Biotin. Hybridized LAMP products and anti-Biotin antibody with a gold label on the conjugated pad of the LFD can form signal or color complexes on the test line. As non-target products were unable to form complexes, no signal or color could be detected [12]. This study aimed to develop LAMP combined with LFD to compare with LAMP-AGE, PCR, and standard *Salmonella* culture as reference methods for detecting *Salmonella* contamination in animal products and animal production environmental samples.

## MATERIALS AND METHODS

### *Salmonella* strains and preparation of the DNA template

*Salmonella* Enteritidis was received from the Department of Veterinary Public Health, Faculty of Veterinary Medicine, Kasetsart University, Thailand (VPHVETKU), as the reference strain for all *Salmonella* spp. strains. The sources of all microorganisms examined are listed in Table 1. The non-*Salmonella* bacterial isolates were obtained from the Microbiology Faculty of Liberal Arts and Science, Kasetsart University, Kamphaeng Sean Campus, Thailand (MICROFLASKU), and the Department of Medical Science Ministry of Public Health, Thailand (DMST). All microorganisms were inoculated with 5 mL of Tryptic Soy Broth (Neogen Company, Lancashire, UK) and cultured for 24 hours at 37°C. For DNA template preparation, 1 mL of bacterial culture was collected into a microcentrifuge tube and centrifuged for 3 min at 10,000 rpm/min. The pellet was washed twice in 1 mL of sterile deionized water after the supernatant was discarded. Subsequently, the pellet was suspended in 100 μL of deionized water and heated at 95°C for 15 min. The supernatant was centrifuged at 12,000 rpm/min for 3 min and transferred to a new tube for the DNA template in LAMP reaction.

### Primers and DNA probes of LAMP-LFD

The *InvA* gene was chosen as the target gene for detecting *Salmonella* in this study, and it was used to design all of the primers. The SalInvA01 primer set shown in the LAMP and PCR tests was derived from Masphol et al [13]. Meanwhile, a pair of PCR primers were used to compare the LAMP assay. The LAMP assay was carried out in 25 μL of reaction mixture containing 1× ThermoPol Buffer, 0.8 μM each of inner primers (SalInvA01-FIP and SalInvA01-BIP), 0.4 μM each of outer primers (SalInvA01-F3 and SalInvA01-B3), 1.4 mM dNTPs, 8 U *Bst* DNA Polymerase (New England Biolabs, Ipswich, MA, USA), 4 mM MgSO_4_, 0.8 M Betaine (Sigma-Aldrich, Darmstadt, Germany), distilled water, and 2 μL of DNA template, as previously described in Masphol et al [13]. The LAMP reaction mixture was incubated for 60 min at 61°C before being terminated at 80°C for 5 min.

The PCR assay was performed in accordance with Masphol et al [13]. In 20 μL of reaction mixture contained 1× PCR Buffer, 2.5 μM of each primer (SalInvA03-F3 and SalInvA03-B3), 2.5 mM dNTPs, 0.3 U of *Taq* DNA polymerase, 4 mM MgCl_2_, distilled water and 2 μL of DNA template. The PCR mixture was pre-denatured at 95°C for 5 min followed by 35 cycles of denaturation at 95°C for 30 seconds, annealing at 61°C for 30 seconds, and extension at 72°C for 30 seconds. The final-extension step was completed at 72°C for 5 min. LAMP and PCR products were electrophoresed in 2% agarose gel, stained with ethidium bromide and viewed under ultraviolet light.

For detection with the LAMP-LFD, the DNA probe was created by combining sequences from the SalInvA01-FIP and SalInvA01-BIP primers. The DIG was labeled using the 5′ end of the SalInvA01-FIP primer, giving rise to the new SalInvA01-DIG primer. Biotin was used to label the 5′ end of the DNA probe (Petty patent submission number 2003 002289). The *InvA* nucleotide sequences in *Salmonella* spp. (GenBank Accession No: DQ644633.1) were aligned with the DNA probe using the CLUSTALW software (https://www.genome.jp/tools-bin/clustalw). All of the LAMP primers or PCR primers and the probe of DNA were synthesized and labeled by (Ward Medic IDT, Bangkok, Thailand).

### LAMP-LFD assay conditions

The LAMP condition used in conjunction with the hybridization processes was modified in line with Masphol et al [13]. This reaction combination was put to the test by varying the amplification temperature, reaction time, and probe concentration. The amplification temperature was adjusted by amplifying the reaction at 61°C, 63°C, and 65°C for 60 min. The reaction time was varied between 30, 40, 50, and 60 min. The probe concentrations of 0.8, 1.6, 3.2, and 4.0 μM were added to the reaction mixture. SalInvA01-DIG and other components were also involved in this reaction. The SalInvA01-DIG for LAMP assay was carried out in 0.8 μM of DIG-labeled SalInvA01-FIP primer, 0.8 μM of SalInvA01-BIP primer, 0.4 μM of SalInvA01-F3 primer, 0.4 μM of SalInvA01-B3 primer, 0.8 M betaine, 1.4 mM each dNTPs, 4 mM MgSO_4_, 8 U of the *Bst* DNA polymerase, 1× ThermoPol Buffer and DNA template 2 μL by the boiling method. After hybridization, LAMP amplicons 8 μL of the LAMP product hybridized with the probe was moved into a new tube containing 100 μL of the Hybri Detect buffer, and the DNA LFD (Serve Science Co., Ltd., Bangkok, Thailand) was dipped in the mixture. The detection result for the test and control lines was detected on the LFD strip within 1 min. For the positive result, two bands were visible on the LFD (one band on the test line and one band on the control line). The sample showed only one band on the control line for the negative result. The test is not functioning correctly if no band is visible on the control line.

### Specificity tests of the LAMP-LFD assays in pure culture

The SP of the LAMP assay was established using 22 isolated bacteria (Table 1). *Salmonella* and non-*Salmonella* strains were tested under optimum conditions using the *InvA* gene. The DNA template of each microbe was amplified by LAMP using SalInvA01-DIG primers. Then, the LAMP product was analyzed with 2% agarose gel electrophoresis and the DNA LFD.

### Limits of detection of the LAMP-LFD and LAMP-AGE assays in pure culture

S. Enteritidis was cultured in tryptic soy broth for 24 hours at 37°C, then diluted ten-fold in sterile water at concentrations ranging from 3×10^1^ CFU/mL to 3×10^8^ CFU/mL (confirmed by viable cells on XLD agar). The DNA template was prepared as described earlier and each of the LAMP products was examined using both agarose gel electrophoresis and the DNA LFD.

### Limits of detection of the LAMP-LFD and LAMP-AGE assays in genomic DNA

The detection limits of the LAMP-LFD and LAMP-AGE assays were determined using genomic DNA from an overnight *Salmonella* pure culture in Tryptic Soy Broth. The genomic DNA was obtained by using the previously described simple boiling method after overnight growth. The Nanodrop 800 spectrophotometer (Thermo Fisher Scientific, Waltham, MA, USA) was used to measure the concentration of DNA at A260/280. And serially diluted 10-fold, each genomic DNA concentration has been used as a DNA template in this LAMP-LFD assay.

### Artificial contamination detection limits in minced chicken with and without pre-enrichment

*S*. Enteritidis was cultured in Tryptic Soy Broth at 37°C for 24 hours before being diluted in sterile water at a concentration range of 3×10^1^ CFU/mL to 3×10^8^ CFU/mL (confirmed by viable cells on XLD agar). The minced chicken was sterilized by autoclaving for use as decontaminated chicken meat. The artificially contaminated food was inoculated with 1 mL of S. Enteritidis at various concentrations into the samples. Then 25 g of artificially contaminated minced chicken sample was homogenized in 225 mL buffer peptone water and incubated at 37°C for 5 hours. After incubation, samples were analyzed at 0 hours (without pre-enrichment) and 5 hours (with pre-enrichment). The DNA of each sample was tested and analyzed using electrophoresis on a 2% agarose gel and the dipstick for DNA lateral flow.

### Detection of *Salmonella* spp. in animal products and animal production environmental samples

#### Sample collection

All the samples were collected within Nakhon Pathom province in Thailand. One hundred twenty animal product samples were sampled and purchased in retail packs from various local markets and supermarkets. Three hundred fifty animal production environmental samples were randomly collected from different farms and slaughterhouses. The samples were collected for determination between June 2020 and July 2021.

#### Sample collection in animal product samples

A total of 120 animal product samples were collected in separate clean plastic bags from 10 samples each of pork, minced pork, chicken meat, minced chicken, beef, chicken balls, pork balls, beef balls. And 20 samples each of chicken cut drumsticks and chicken cut fillets. All samples were tested immediately after purchase and transferred in an icebox to a laboratory.

#### Sample collection in animal production environmental samples

A total of 350 samples were obtained from different areas. In swine housing, 60 samples were collected from 20 swab samples each of the floor in a dry area, feed bunks, and the floor in a wet area. In poultry housing, 60 samples were collected from 20 swab samples each of the floor, feeder, and cage wire mesh. In milking parlor, 60 samples were collected from 20 swab samples each of the teat wipe towels, floor, and milking unit. In feed bulk truck, 60 samples were collected from 20 swab samples each of the feed conveyor, feed tank, and wheel. In beef slaughterhouse, 60 samples were collected from 20 swab samples each of the butcher table, knife, and floor. In poultry slaughterhouse, 50 samples were collected from 10 swab samples each of the chiller water, floor, conveyor belt, cutting board and knife. Sterilized cotton swabs (1 swab/sample) dipped in 10 mL of phosphate-buffered saline in sterile conical tubes with caps were used to swab and collect samples from the exposed surface area of 100 cm^2^. All samples were sent to the laboratory on ice and were analyzed.

#### Sample preparation and analysis

In animal product samples, 25 g of animal product samples were weighed and sliced into small pieces by using a sterile scalpel and forceps on a sterile petri dish. Then, it was placed in a sterile bag with 225 mL of buffer peptone water. For environmental samples from animal production, each animal production environmental sample swab was vortex mixed and homogenized to aid the release of organisms into the diluent. The diluent sample swab was added into a sterile bag containing 225 mL of buffer peptone water. Then, each animal product sample and the animal production environmental samples in a sterile bag with 225 mL of buffer peptone water were homogenized by using a food stomacher for 2 min before pre-enrichment step. For 5 hours; the DNA template of each sample was prepared as described earlier and analyzed using LAMP-AGE, LAMP-LFD, and PCR assays. For the culture method, it was continuously incubated at 37°C for 24 hours. *Salmonella* spp. was isolated according to an international standard culture method (Including WHO 2010 for detecting *Salmonella*) [14]. Following pre-enrichment, 1 mL aliquots of samples were added to 9 mL of Mueller Kauffman Tetrathionate Novobiocin Broth Base (HiMedia Laboratories, Mumbai, India), and incubated at 37°C for 24 hours, while 0.1 mL aliquots of samples were added to 9.9 mL Rappaport Vassiliadis Soya Broth (HiMedia Laboratories, India), and incubated at 42°C for 24 hours. A 10-μL loop full of selective enrichment was spread and incubated overnight at 37°C for each selective plating on Xylose Lysine Deoxycholate Agar (HiMedia Laboratories, India) and Brilliant Green Agar (HiMedia Laboratories, India). *Salmonella* colonies were selected and transferred to tubes for biochemical identification from each selective agar.

### Kappa statistics and accuracy analysis

The performance indicators for qualitative microbiological methods were calculated accuracy (AC), sensitivity (SE), specificity (SP), positive predictive value (PPV). The degree of agreement between the methods tested, as described by using Cohen's kappa (k), according to previously study [15] that described the kappa index was used: perfect agreement (1.00), nearly perfect agreement (0.81 to 0.99), substantial agreement (0.61 to 0.80), moderate agreement (0.41 to 0.60), fair agreement (0.21 to 0.40), slight agreement (0.01 to 0.20), and less than a chance agreement (<0).

## RESULTS

### Optimization of LAMP-LFD reaction conditions

**Figure 1:**
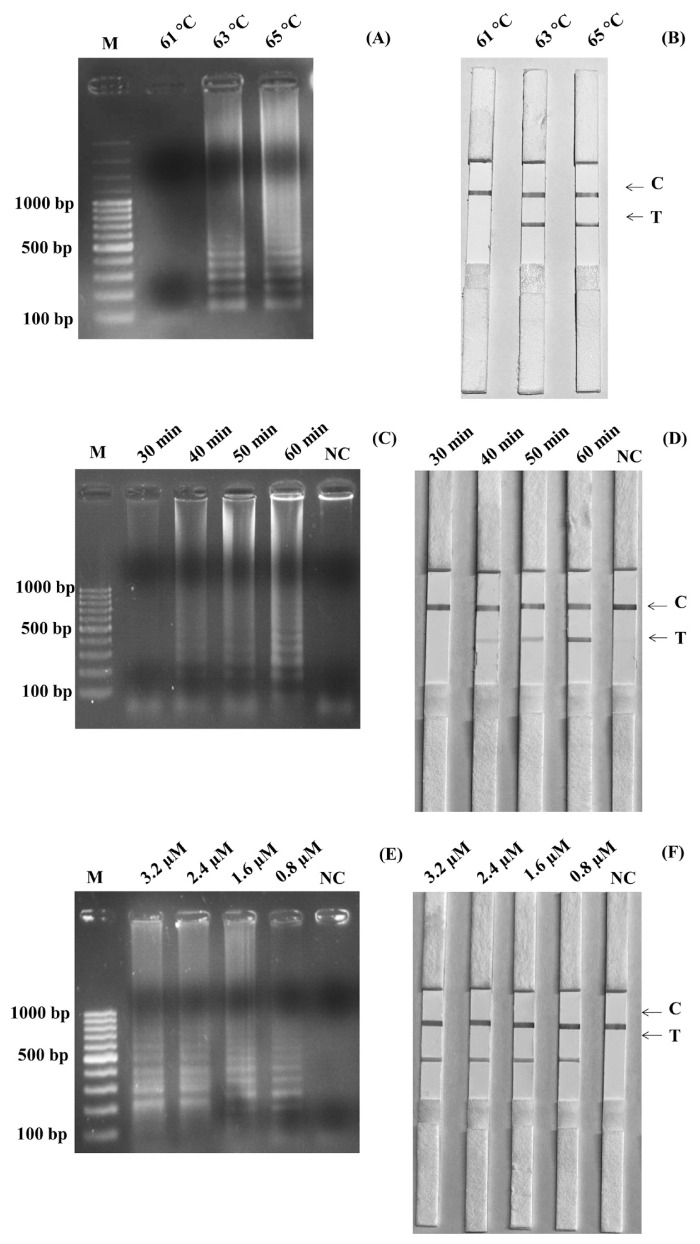
Optimization of SalInvA01-DIG LAMP reaction temperature. The LAMP-LFD assay were optimized temperature and assessed based on 2% agarose gel electrophoresis (A) and lateral flow dipstick (B) negative control without DNA template (NC), control line (C), and test line (T). LAMP-LFD, loop-mediated isothermal amplification combined with lateral flow dipstick.

The optimized temperature of the SalInvA01-DIG primer was between 63°C and 65°C. The agarose gel electrophoresis results revealed that 63°C showed the cleanest bands with a ladder-like pattern of DNA. The optimal temperature for the LAMP-LFD reaction was chosen as 63°C in Figure 1A. In the LAMP-LFD study, LFD analysis revealed cherry-red bands in both the test and control lines at 63°C and 65°C that 63°C and both the test and control lines had red bands at 65°C, as well as a cherry-red band in the control line at 61°C (Figure 1B). The SalInvA01-DIG primer amplified the DNA ladder band at 40 min but dense and clear bands appeared at 60 min (Figures 1C, 1D). The optimized LAMP reaction time for the SalInvA01-DIG primer was 60 min. For concentrations of the probe, the result showed that 0.8 μM of DNA probe provided the lowest concentration while still producing a cherry-red color on the test line of the LFD (Figures 1E, 1F).

### The specificity of the LAMP-LFD reaction

**Figure 2:**
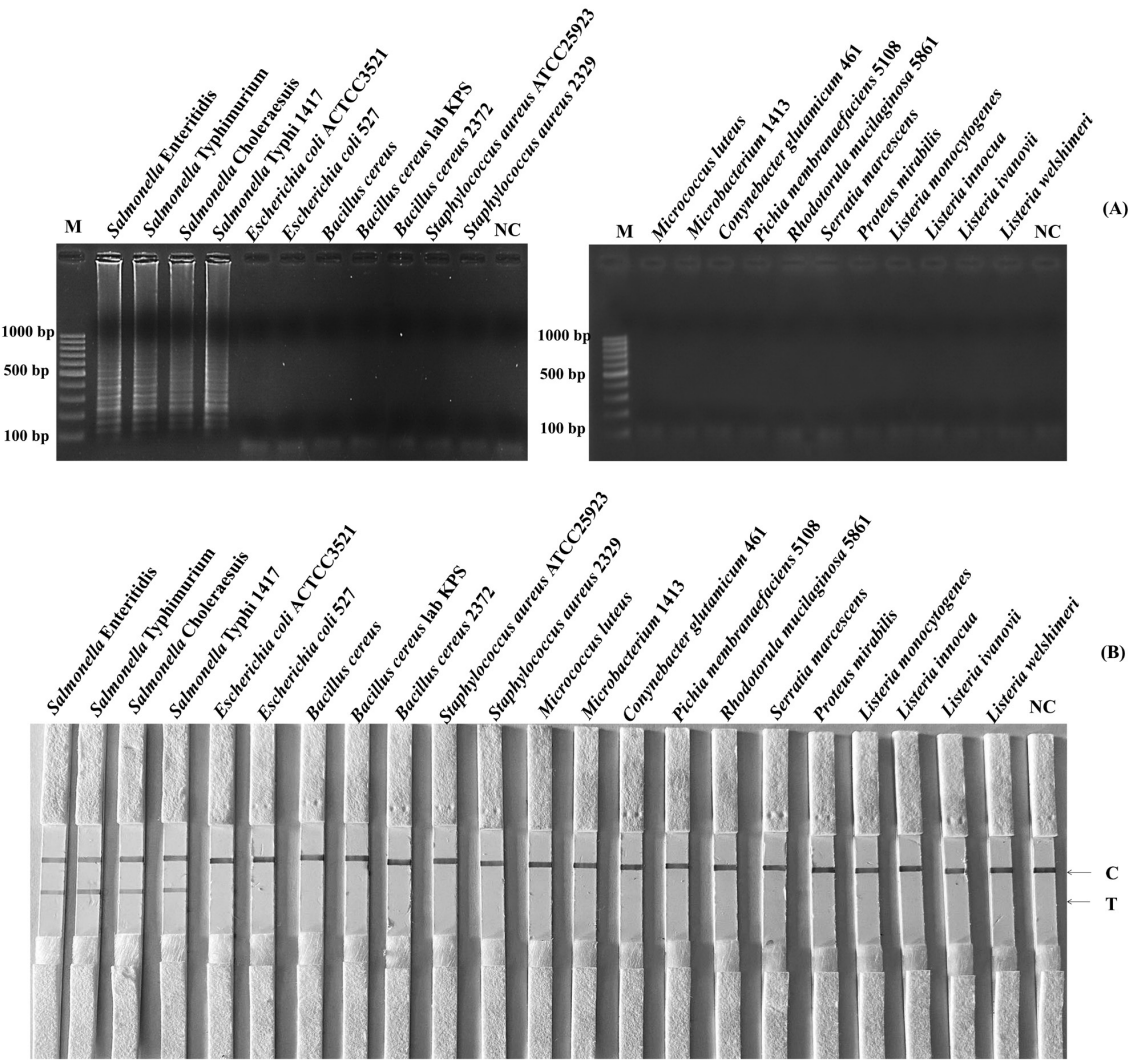
The specificity test of SalInvA01-DIG primer sets using LAMP-LFD, Lane NC represents negative control (without DNA template), control line (C) and test line (T). LAMP-LFD, loop-mediated isothermal amplification combined with lateral flow dipstick.

The LAMP-AGE tests are used to determine the SP in Figure 2A, while the LAMP-LFD assays are shown in Figure 2B for *Salmonella* detection utilizing SalInvA01-DIG primer sets are designed to specifically target *InvA* genes. The result demonstrated high SP by amplifying only 4 strains of *Salmonella* included *S*. Typhimurium, *S*. Enteritidis, *S*. Choleraesuis and *S*. Typhi 1417 from other microbial isolates such as *E. coli*, *E. coli 527*, *B. cereus*, *B. cereus* lab KPS, *B. cereus* 2372, *S. aureus*, *S. aureus* 2329, *Micrococcus luteus*, *Microbacterium* 1413, *Conynebacter glutamicum* 461, *Pichia membranaefaciens* 5108, *Rhodotorula mucilaginosa* 5861, *Serratia marcescens*, *Proteus mirabilis*, *L. monocytogenes*, *L. ivanovii*, *L. innocua*, and *L. welshimeri*, all of which did not show any cross-reactivity.

### Limit of detection in pure culture

**Figure 3:**
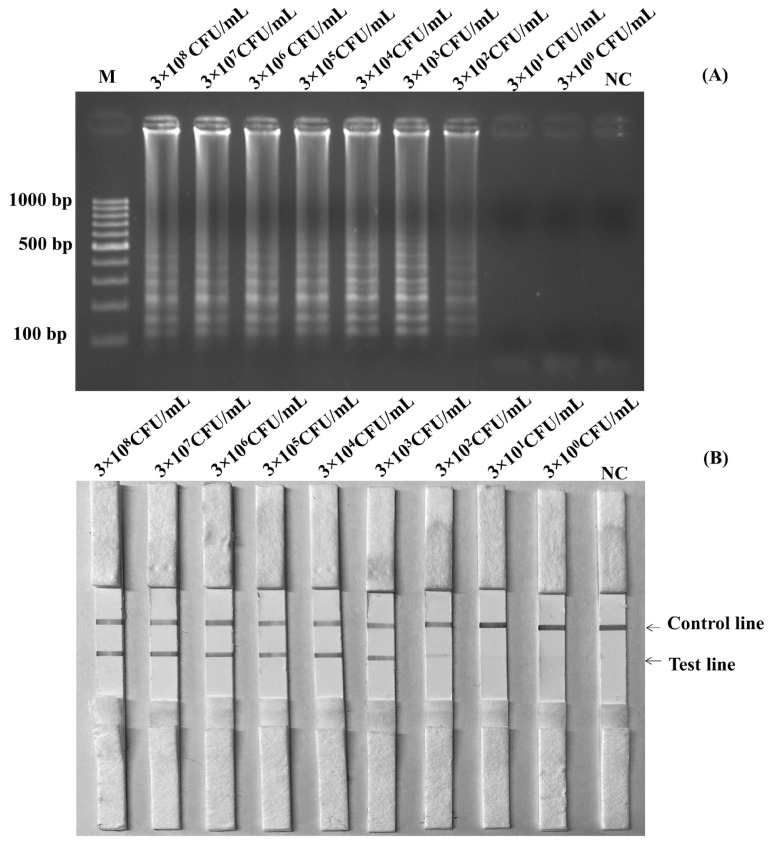
Limit of detection (LOD) for detection of *Salmonella* spp. in pure culture by using LAMP-AGE (A) and LAMP-LFD (B). Lane M represents 100 bp DNA ladder marker, Lane NC represents negative control (without DNA template). LAMP-LFD, loop-mediated isothermal amplification combined with lateral flow dipstick.

Before using the boiling DNA preparation method and amplification, cultures of *S*. Enteritidis containing 3×10^8^ CFU/mL were diluted to be 3×10^7^ CFU/mL, 3×10^6^ CFU/mL, 3×10^5^ CFU/mL, 3×10^4^ CFU/mL, 3×10^3^ CFU/mL, 3×10^2^ CFU/mL, and 3×10^1^ CFU/mL. Figure 3A and Figure 3B showed the results of the limits of detection for the LAMP-LFD and LAMP-AGE tests in pure culture. The detection of limits using the agarose gel electrophoresis and the LFD was found at 3×10^2^ CFU/mL or 6 CFU/reaction.

### The detection limit of the LAMP-LFD and LAMP-AGE assays in genomic DNA

**Figure 4:**
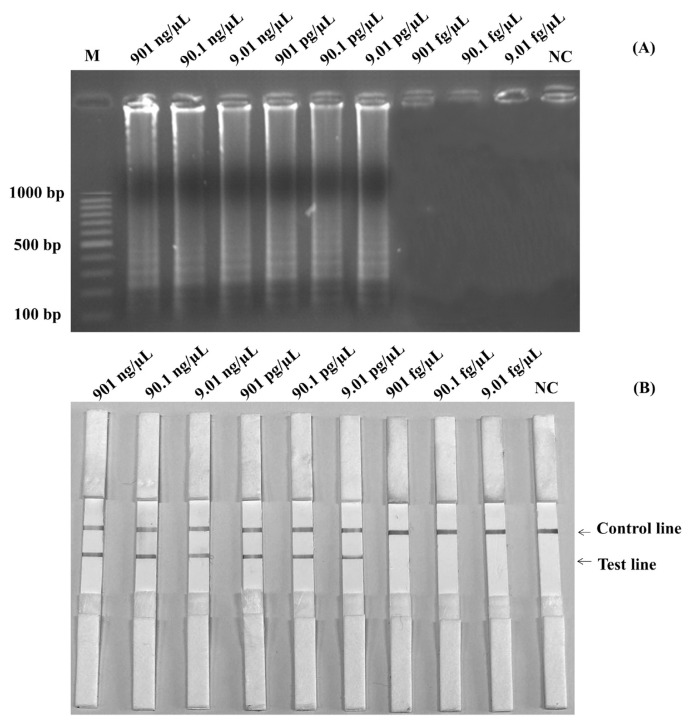
Limit of detection (LOD) for detection of purified DNA of *Salmonella* by using LAMP-AGE (A) and LAMP-LFD (B). Lane M represents 100 bp DNA ladder marker, Lane NC represents negative control (without DNA template). Lane M represents 100 bp DNA ladder marker, Lane NC represents negative control (without DNA template). LAMP-LFD, loop-mediated isothermal amplification combined with lateral flow dipstick.

The initial genomic DNA concentration was measured at 901 ng/μL. To establish the lowest genomic DNA concentration required for the SalInvA01-DIG primer, the detection limit of genomic DNA was 9.01 pg/μL based on the *InvA* gene. The results are shown by LAMP-AGE in Figure 4A and LAMP-LFD in Figure 4B.

### Limits of detection in artificially contaminated minced chicken with and without pre-enrichment

**Figure 5:**
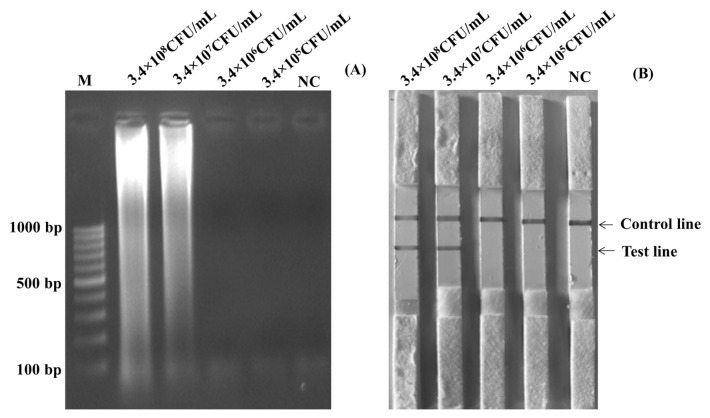
Limit of detection (LOD) for detection of *Salmonella* in artificial contamination chicken without pre-enrichment by using LAMP-AGE (A) and LAMP-LFD (B). Lane M represents 100 bp DNA ladder marker, Lane NC represents negative control (without DNA template). LAMP-LFD, loop-mediated isothermal amplification combined with lateral flow dipstick.

**Figure 6:**
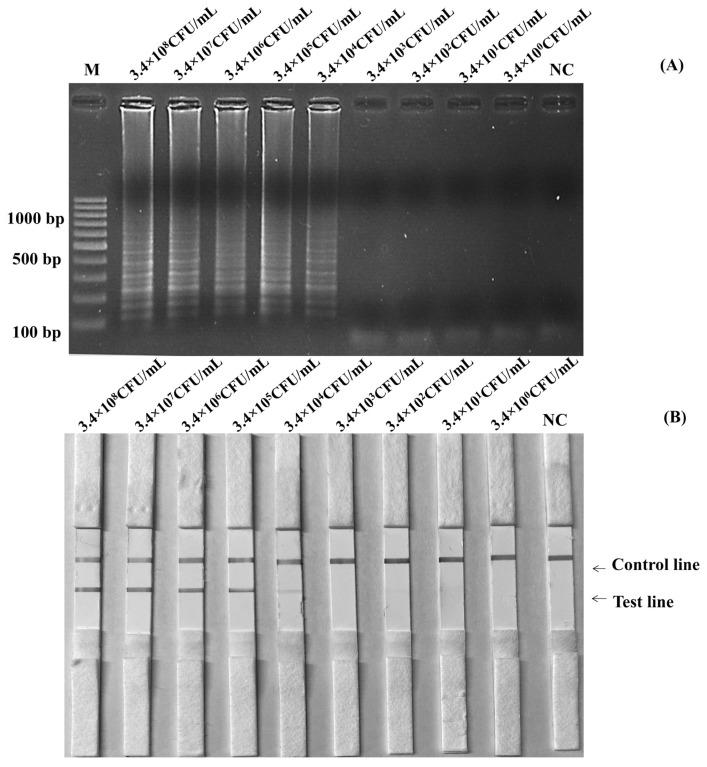
Limit of detection (LOD) for detection of *Salmonella* in artificial contamination chicken with pre-enrichment by using LAMP-AGE (A) and LAMP-LFD (B). Lane M represents 100 bp DNA ladder marker, Lane NC represents negative control (without DNA template). LAMP-LFD, loop-mediated isothermal amplification combined with lateral flow dipstick.

All autoclaved chicken meat samples containing 25 g in 225 mL of buffer peptone water were inoculated with *S*. Enteritidis, giving a positive result. The sample of non-inoculated chicken meat tested negative. The *Salmonella* detection limit in LAMP-LFD without pre-enrichment testing of artificially contaminated chicken meat samples was 3.4×10^7^ CFU/mL (Figure 5). After incubating the chicken meat samples at 37°C for 5 hours with pre-enrichment, the limit of detection increased to 3.4×10^4^ CFU/mL or 680 CFU/reaction (Figure 6).

### Detection of *Salmonella* spp. in the animal products and animal production environmental samples

**Figure 7:**
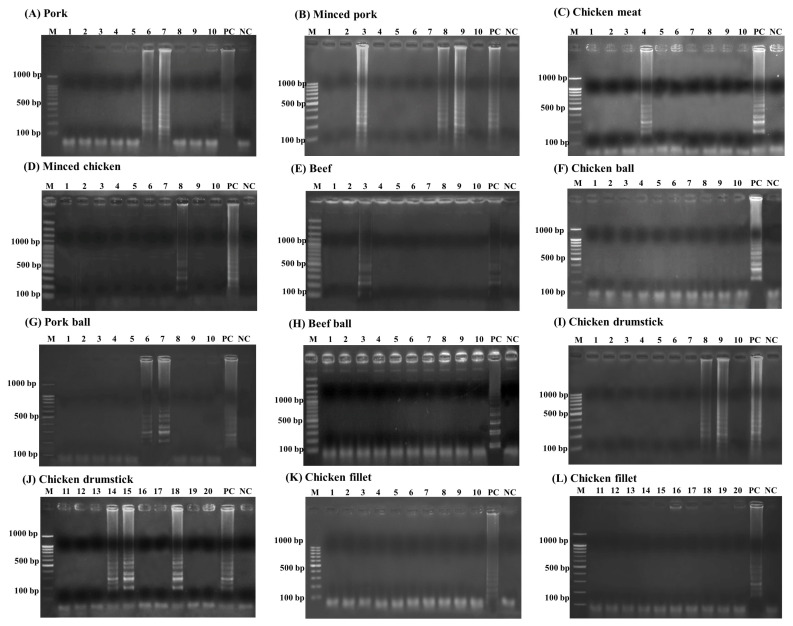
The detection results of *Salmonella* in animal products using LAMP-AGE. (A) pork, (B) minced pork, (C) chicken meat, (D) minced chicken, (E) beef, (F) chicken ball, (G) pork ball, (H) beef ball, (I–G) chicken drumstick and (K–L) chicken fillet. Lane M represents 100 bp DNA ladder marker, Lane PC represents positive control Lane NC represents negative control (without DNA template).

**Figure 8:**
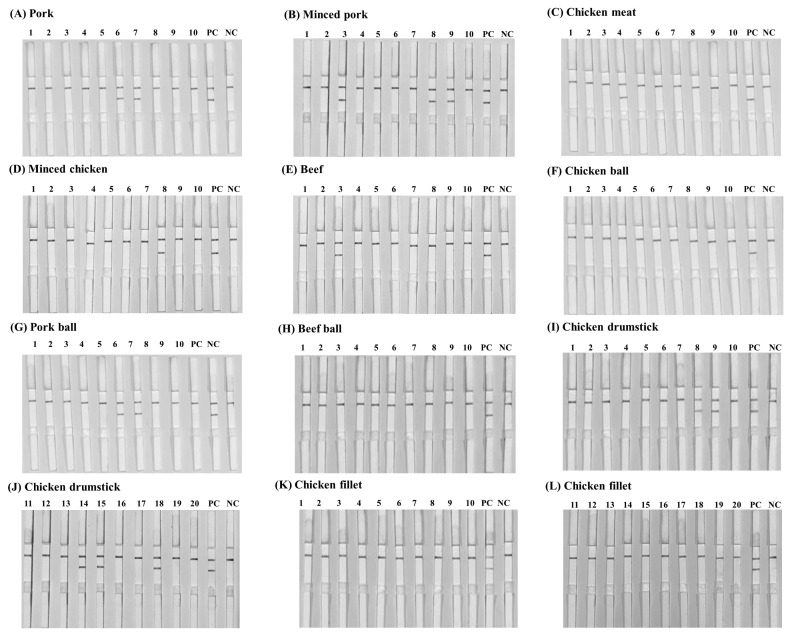
The detection results of *Salmonella* in animal products using LAMP-LFD. (A) pork, (B) minced pork, (C) chicken meat, (D) minced chicken, (E) beef, (F) chicken ball, (G) pork ball, (H) beef ball, (I–G) chicken drumstick and (K–L) chicken fillet. PC represents positive control NC represents negative control (without DNA template). LAMP-LFD, loop-mediated isothermal amplification combined with lateral flow dipstick.

**Figure 9:**
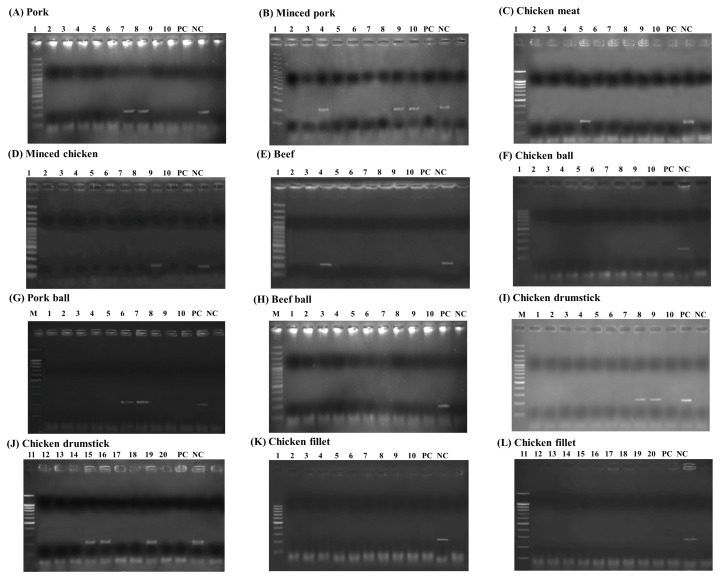
The detection results of Salmonella in animal products using PCR assay. (A) pork, (B) minced pork, (C) chicken meat, (D) minced chicken, (E) beef, (F) chicken ball, (G) pork ball, (H) beef ball, (I–G) chicken drumstick and (K–L) chicken fillet. Lane M represents 100 bp DNA ladder marker, Lane PC represents positive control Lane NC represents negative control (without DNA template). LAMP-LFD, loop-mediated isothermal amplification combined with lateral flow dipstick.

Among these 120 animal products, the LAMP-AGE, LAMP-LFD, PCR, and culture methods were evaluated. This study examined 10 samples each of pork, minced pork, chicken meat, minced chicken, beef, chicken balls, pork balls, beef balls, and 20 samples each of chicken cut drumsticks and chicken cut fillets. The results of *Salmonella* detection in the animal products showed 15 samples had positive results using LAMP-LFD, LAMP-AGE, and PCR in Figures 7 to 9. Table 2 shows that 10/120 (8.3%) of all culture method, LAMP-LFD, LAMP-AGE, and PCR samples tested positive for *Salmonella*. For pork samples, 1/10 positive samples were detected with the culture method, while LAMP-LFD, LAMP-AGE, and PCR showed *Salmonella* in 2/10 samples. Minced pork (3/10), chicken meat (1/10), minced chicken (1/10), and beef (1/10) were positive for *Salmonella*, using four methods. The chicken balls (0/10), pork balls (0/10), beef balls (0/10) and chicken cut fillets (0/10) tests for *Salmonella* were negative and detected by all four methods. Five false positive samples [pork balls (2/10), chicken cut drumsticks (3/20)] were detected in this study using LAMP-LFD, LAMP-AGE, and PCR.

The total of 350 animal production environmental samples were examined by 60 samples of swine housing, poultry housing, milking parlor, feed bulk truck, beef slaughterhouse, and 50 samples of poultry slaughterhouse. The results shown in Table 3 reveal that 91/350 (26%), 91/350 (26%), 91/350 (26%), 86/350 (24.6%) samples examined by the culture method, LAMP-LFD, LAMP-AGE, and PCR respectively were positive for *Salmonella*. In swine housing, the culture method, LAMP-LFD, and LAMP-AGE detected *Salmonella* in the floor (dry area) (7/20), and each of feed bunk and floor (wet area) (8/20) samples, while (7/20) each of floor (dry area), feed bunk and floor (wet area) were detected with PCR. For poultry housing, the culture method, LAMP-LFD, and LAMP-AGE detected *Salmonella* in the floor (8/20), feeder (9/20) and cage wire mesh (7/20) samples, while (8/20) floor, (7/20) each of feeder and cage wire mesh samples were detected with PCR. For beef slaughterhouse, the culture method, LAMP-LFD, and LAMP-AGE detected *Salmonella* in the butcher table (3/20), knife (1/20) and floor (4/20) samples, while (1/20) knife, (3/20) and each of butcher table and floor samples were detected with PCR. Milking parlor, teat wipe towels (5/20), floor (4/20), and milking unit (4/20) were positive for *Salmonella* and were discovered by the four methods. Feed bulk truck, feed conveyor (2/20), feed tank (1/20), and wheel (4/20) were positive for *Salmonella* and were found using all four methods. In the poultry slaughterhouse, chiller water (3/10), floor (4/10), conveyor belt (4/10) cutting board (2/10), and knife (3/10) produced positive results for *Salmonella* and were detected by the four methods. No false negative result was observed in either LAMP-LFD or LAMP-AGE. Five false negative samples (feed bunk [1/20], floor [wet area] [1/20], feeder [2/20] and floor [beef slaughterhouse] [1/5]) were found using PCR. Eleven false positive samples (feed bunk [1/20], floor [poultry housing] [1/20], cage wire mesh [3/20], teat wipe towels [2/20], knife [poultry slaughterhouse] [1/20], and each of chiller water, conveyor belt and cutting board [1/10]) were detected in this study using three methods.

### Kappa index and accuracy

The SE, SP, PPV, AC, and Kappa Index of LAMP-LFD, LAMP-AGE, PCR, compared with culture method were determined and showed in Table 4. The SE, SP, PPV, and AC for LAMP-LFD and LAMP-AGE were 100%, 95.9%, 87.2%, and 96.8%, respectively. Kappa Index was 0.90, indicating nearly perfect agreement with culture method. While, PCR method, The SE, SP, PPV, and AC were 95.0%, 95.7%, 85.7%, and 95.5%, respectively. Kappa Index was 0.88, indicating nearly perfect agreement with culture method.

## DISCUSSION

The *InvA* gene is the most commonly utilized and successful gene for generating LAMP primers to detect *Salmonella* [12]. LAMP was originally presented for *Salmonella* detection in 2005. Later, modified *Salmonella* diagnosis methods based on various target genes were developed, such as *stn*, *hilA*, *bcfD*, and *fimY* [13]. LAMP products have been detected using a variety of approaches. The LFD assay provides good SP testing and visual result evaluation [12,16]. In the previous study, the researchers developed the LAMP technique in conjunction with the LFD technique to examine and report the results. In this study, the LAMP technique in combination with the LFD technique (LAMP-LFD) was selected based on the *InvA* gene and achieved the best primer set from previous studies (Masphol et al [13]). A modified LAMP product analysis protocol from gel electrophoresis and the addition of SYBR Green I to the LFD for detecting *Salmonella* spp. Also, new probe was designed to target the *InvA*. labeled DIG-with FIP primers and Biotin-labeled DNA probe, primer set and DNA probe labeled was used for the LAMP technique in conjunction with the LFD technique (LAMP-LFD) and focused on the LAMP-LFD technique to examine *Salmonella* contamination compared to other methods such as LAMP-AGE, PCR, and culture methods. For LAMP-LFD, a reaction temperature of 63°C for 60 min and an increased probe concentration of 0.8 μM within the LAMP reaction are required. Hybridization of the probe is used in most of the studies that follow the LAMP reaction [1,5]. Probes were hybridized along with the LAMP method, which was used in this study. It suggests that hybridizing a probe with the LAMP reaction shortens the step of the LAMP combination in the LFD reaction.

The LAMP-LFD method for *Salmonella* targeting the *InvA* gene was effectively developed in this study. The SalInvA01-DIG primer set has no cross-reactivity with target genes in non-*Salmonella* strains. The result shows that the LAMP-LFD assays based on the SalInvA01-DIG primer set of *InvA* gene are very efficient and specific for detecting *Salmonella* spp. According to the same protocol as in the previous study [13], the genomic DNA of each of the 19 microorganisms was tested using the fluorescent dyes (LAMP-SYBR green I) and agarose gel electrophoresis (LAMP-AGE). This included *S*. Typhimurium, *S*. Enteritidis, *S*. Typhi, *E. coli*, *E. coli* 527, *B. cereus*, *B. cereus* lab KPS, *B*. cereus 2372, *L. monocytogenes*, *S. aureus*, *S. aureus* 2329, *Micrococcus luteus*, *Microbacterium* 1413, *Corynybacter glutamicum* 461, *Pichia membranaefaciens* 5108, *Rhodotorula mucilaginosa* 5861, *Serratia marcescens*, and *Proteus mirabilis*.

In this study, the limit of detection based on LAMP-LFD for *Salmonella* pure culture was 3×10^2^ CFU/mL while genomic DNA was 9.01 pg/μL. In previous studies, the *Salmonella* genomic DNA detection limit targeting similar gene with our work in the *InvA* gene was 89 fg/μL [12]. For *Salmonella* targeting different gene with this work, in the *hilA* gene was 6.7 CFU/mL in pure culture and 13.5 fg/μL in genomic DNA [17]. And *Salmonella* cells targeting the *siiA* gene was 3.7 CFU/mL [18]. This result indicated that the efficiency of amplification may be affected by the primers used for different genes, or even the same target gene. It may lead to a diverse range of detection limits [17]. Moreover, Liu et al [12] reported the LAMP-LFD was repeatable and obtained identical findings at 8.9 pg/μL. This study found that LAMP with a hybridized probe has a better limit of detection than LAMP because the probe may increase the LAMP reaction by hybridizing to the LAMP product during the LAMP reaction. Similarly Kumvongpin et al [19] and Augkarawaritsawong et al [20] demonstrated increasing of the probe and limit of detection.

In the case of artificial contamination of the chicken sample, the *Salmonella* detection limits used by LAMP-LFD with pre-enrichment were 3.4×10^4^ CFU/mL (680 CFU/reaction), and 3.4×10^7^ CFU/mL without pre-enrichment. The detection limit of *Salmonella* spp. in pure culture was higher than *Salmonella* spp. in minced chicken samples. This is because the chicken samples may contain inhibitors found in the food sample such as fats, proteins, enzymes or other compounds that affect the detection limit of *Salmonella* spp. [13]. The enrichment step is a procedure to increase the SE of pathogen analysis by increasing the number of target microorganisms in low or susceptible conditions. The sample is then processed for further analysis using standard methods such as culture, PCR, and LAMP. This demonstrates that the enrichment procedure ensures enough SE to be correct and accurate [21]. Previous studies have discovered that LAMP-LFD has a detection limit equal to or lower than this study, based on the *InvA* gene: 8×10^4^ CFU/25 g in artificially contaminated chicken feed [12] and 1×10^4^ CFU/mL in milk [22]. Another study discovered various genes at, 2.2 CFU/mL in powdered infant formula based on the *siiA* gene [18], and 1.44 CFU/mL in the *hilA* gene-based food matrix (raw milk, pork, beef, and chicken meat) [17]. According to several governmental regulations, live *Salmonella* must not be detectable in 25 g of food. The limit of detection (corresponds to 3×10^4^ CFU/mL) does not meet the requirement for quantification of low concentration, but is sufficient for qualitative detection. Despite the effectiveness of the LAMP-LFD method, it still needs to be developed, popularized, and used in public health and food detection [23].

The LAMP-LFD, LAMP-AGE, PCR, and culture method were evaluated and compared for their AC, SE, SP, PPV, and Kappa index. LAMP-LFD and LAMP-AGE showed excellent SE, SP, PPV, AC, and Kappa Index more than PCR at 100%, 95.9%, 87.2%, and 96.8%, respectively. For Kappa index of LAMP-LFD was 0.90, indicated nearly perfect agreement with culture method. It was found that the result obtained from this study were better than previous [24]. In another study, Hyeon et al [25] reported that the label-based LFD with multiplex LAMP detected *Salmonella* spp. and *Cronobacter* spp. in powdered infant formula (n = 80) and showed 100% SP in this method. Among the animal products, the most positive for *Salmonella* was found to be minced pork (3/10) and chicken cut drumsticks (3/20) by using the four methods. Minced meat contains fat, which can protect *Salmonella* from the environment. Other food samples lack these qualities and their surface dries rapidly making them unsuitable for *Salmonella* [26]. For chicken cut drumsticks, which are bone-in products, a previous study suggested that internalized *Salmonella* through bone could be the cause of disease in these products [27]. Almost all detection methods are consistent, including LAMP-LFD, LAMP-AGE, PCR, and the culture method. As with LAMP-LFD, closer DNA-based detection methods like PCR and other comparable assays produce *Salmonella* detection results which are not always totally compatible with the culture method. Furthermore, PCR and LAMP can identify non-viable *Salmonella* as well as viable but non-culturable *Salmonella* as a reaction to poor environmental conditions [17]. This was also shown in this study, particularly for swabs acquired from the floor and cage wire mesh, as the samples were swabbed after being cleaned with powerful chemical detergents. The characteristics of methods based on nucleic acid amplification may also lead to false-positive examination results for the culture method. For example, knives and cutting boards produced false positive results. Because it is possible that DNA from *Salmonella* cells that have died will be amplified, which would explain the increased *Salmonella* molecular positive in culture-negative samples [28,29]. The enrichment has been incubated for 5 hours rather than overnight in the culture method, the incubation time may be insufficient to ensure sub-lethally dead cells are revived or resurrected. Despite most reports of improved molecular testing methods and enhanced sample processing efficiency, enrichment is still required [30]. This is because low concentrations are possible and cause sub-lethal wounds to the target, as well as a large number of other bacteria. Non-culturable *Salmonella* or incubation times that are too short may reduce diagnosis SE, resulting in false negative results [31]. This is especially true for chilled water, which may be contaminated with background microbiota that would inhibit *Salmonella* growth. This LAMP-LFD assay has several benefits that make it valuable in conditions where time and resources are limited. First, the LAMP-LFD reaction does not necessitate the use of specialist equipment includes PCR machine and gel electrophoresis systems. Second, LAMP-LFD reactions could be carried out at an isothermal temperature of 60°C to 65°C. Alternatively, the PCR necessitates strict temperature control during pre-denaturing, denaturing, annealing, extension, and final-extension. Third, the LAMP-LFD assay is a diagnostic tool that saves time. It only takes 1.30 hours to detect LAMP and LFD using the two-step assay. The LAMP-AGE reaction is also a two-step assay that takes 2 hours to amplify by LAMP and analyze by agarose gel electrophoresis. Meanwhile, PCR reactions are usually doubled by LAMP-LFD. Fourth, the fluorometer is not required to monitor the fluorescent signal in the LAMP-LFD assay. LAMP results were seen with the naked eye on the dipsticks, making this platform easier to use than others.

In conclusion, The LAMP-LFD assay was developed to detect *Salmonella* with accuracy, simplicity, and rapidity. The *InvA* gene was used to determine the specific primer combination. The LAMP-LFD assay could also be completed in 1.30 hours at a temperature of 63°C. This assay offers notable benefits. Only the primer set and a DNA probe are utilized to identify the amounts of target DNA. Meanwhile, the LAMP amplicons could produce visible lines on LFDs without the need for gel electrophoresis. Furthermore, SE studies found that the LAMP-LFD assay was capable of detecting as low a level as 9.01 pg/μL of genomic DNA of *Salmonella*. Furthermore, the LAMP-LFD assay detected *Salmonella* at higher rates in animal products and animal production environmental samples than the LAMP-AGE and PCR. This LAMP-LFD assay has high potential for use as a primary *Salmonella* screening assay in animal products and animal production environmental samples.

## Figures and Tables

**Table 1:** Bacterial strains used for assays

Species	Source^1)^
*Salmonella* species	
* Salmonella* Typhimurium	VPHVETKU
* Salmonella* Enteritidis	VPHVETKU
* Salmonella* Choleraesuis	VPHVETKU
* Salmonella* Typhi 1417	VPHVETKU
Non-Salmonella species	
* * *L. innocua*	DMST
* * *L. ivanovii*	DMST
* * *L. welshimeri*	DMST
* * *L. monocytogenes*	VPHVETKU
* Escherichia coli* ATCC3521	VPHVETKU
* Escherichia coli* 527	VPHVETKU
* * *Bacillus cereus*	MICROFLASKU
* Bacillus cereus* lab KPS	MICROFLASKU
* Bacillus cereus* 2372	MICROFLASKU
* Staphylococcus aureus* ATCC25923	MICROFLASKU
* Staphylococcus aureus* 2329	MICROFLASKU
* * *Micrococcus luteus*	MICROFLASKU
* Microbacterium* 1413	MICROFLASKU
* Conynebacter glutamicum* 461	MICROFLASKU
* Pichia membranaefaciens* 5108	MICROFLASKU
* Rhodotorula mucilaginosa* 5861	MICROFLASKU
* * *Serratia marcescens*	MICROFLASKU
* * *Proteus mirabilis*	MICROFLASKU

1) VPHVETKU, Veterinary Public Health, Veterinary Medicine, Kasetsart University; DMST, Department of Medical Sciences Thailand; MICROFLASKU, Microbiology Faculty of Liberal Arts and Science, Kasetsart University.

**Table 2:** Detection of *Salmonella* spp. in animal product using by culture method, LAMP-AGE, LAMP-AGE, and PCR^1)^

Animal product samples	Number of samples/number of total samples (%)^2)^									

	Culture method	LAMP-LFD			LAMP-AGE			PCR		
			
	Positive	TP	FP	FN	TP	FP	FN	TP	FP	FN
Pork	1/10 (10)	2/10 (20)	0/10 (0)	0/10 (0)	2/10 (20)	0/10 (0)	0/10 (0)	2/10 (20)	0/10 (0)	0/10 (0)
Minced pork	3/10 (30)	3/10 (30)	0/10 (0)	0/10 (0)	3/10 (30)	0/10 (0)	0/10 (0)	3/10 (30)	0/10 (0)	0/10 (0)
Chicken meat	1/10 (10)	1/10 (10)	0/10 (0)	0/10 (0)	1/10 (10)	0/10 (0)	0/10 (0)	1/10 (10)	0/10 (0)	0/10 (0)
Minced chicken	1/10 (10)	1/10 (10)	0/10 (0)	0/10 (0)	1/10 (10)	0/10 (0)	0/10 (0)	1/10 (10)	0/10 (0)	0/10 (0)
Beef	1/10 (10)	1/10 (10)	0/10 (0)	0/10 (0)	1/10 (10)	0/10 (0)	0/10 (0)	1/10 (10)	0/10 (0)	0/10 (0)
Chicken balls	0/10 (0)	0/10 (0)	0/10 (0)	0/10 (0)	0/10 (0)	0/10 (0)	0/10 (0)	0/10 (0)	0/10 (0)	0/10 (0)
Pork balls	0/10 (0)	0/10 (0)	2/10 (20)	0/10 (0)	0/10 (0)	2/10 (20)	0/10 (0)	0/10 (0)	2/10 (20)	0/10 (0)
Beef balls	0/10 (0)	0/10 (0)	0/10 (0)	0/10 (0)	0/10 (0)	0/10 (0)	0/10 (0)	0/10 (0)	0/10 (0)	0/10 (0)
Chicken cut drumsticks	3/20 (15)	2/20 (15)	3/20 (10)	0/20 (0)	2/20 (15)	3/20 (10)	0/20 (0)	2/20 (15)	3/20 (10)	0/20 (0)
Chicken cut fillets	0/20 (0)	0/20 (0)	0/20 (0)	0/20 (0)	0/20 (0)	0/20 (0)	0/20 (0)	0/20 (0)	0/20 (0)	0/20 (0)
Overall (n = 120)	10/120 (8.3)	10/120 (8.3)	5/120 (4.2)	0/120 (0)	10/120 (8.3)	5/120 (4.2)	0/120 (0)	10/120 (8.3)	5/120 (3.34.2)	0/120 (0)

LAMP-LFD, loop-mediated isothermal amplification combined with lateral flow dipstick; PCR, polymerase chain reaction.

1) Culture method was regarded as reference method.

2) TP, true positive: samples detected as positive using LAMP-LFD or LAMP-AGE or PCR were also positive using the reference method; FP, false positive: samples detected as positive using LAMP-LFD or LAMP-AGE or PCR, but negative by the reference method; FN, false negative: samples detected as negative using LAMP-LFD or LAMP-AGE or PCR, but positive by the reference method.

**Table 3:** Detection of *Salmonella* spp. in animal production environmental samples using culture method, LAMP-LFD, LAMP-AGE, and PCR^1)^

Environmental samples	Number of samples/Number of total samples (%)^2)^									

	Culture method	LAMP-LFD			LAMP-AGE			PCR		
			
	Positive	TP	FP	FN	TP	FP	FN	TP	FP	FN
Swine housing										
Floor (dry area)	7/20 (35)	7/20 (35)	0/20 (0)	0/20 (0)	7/20 (35)	0/20 (0)	0/20 (0)	7/20 (35)	0/20 (0)	0/20 (0)
Feed bunk	8/20 (40)	8/20 (40)	1/20 (5)	0/20 (0)	8/20 (40)	1/20 (5)	0/20 (0)	7/20 (35)	1/20 (5)	1/20 (5)
Floor (wet area)	8/20 (40)	8/20 (40)	0/20 (0)	0/20 (0)	8/20 (40)	0/20 (0)	0/20 (0)	7/20 (35)	0/20 (0)	1/20 (5)
Poultry housing										
Floor	8/20 (40)	8/20 (40)	1/20 (5)	0/20 (0)	8/20 (40)	1/20 (5)	0/20 (0)	8/20 (40)	1/20 (5)	0/20 (0)
Feeder	9/20 (45)	9/20 (45)	0/20 (0)	0/20 (0)	9/20 (45)	0/20 (0)	0/20 (0)	7/20 (35)	0/20 (0)	2/20 (10)
Cage wire mesh	7/20 (35)	7/20 (35)	3/20 (15)	0/20 (0)	7/20 (35)	3/20 (15)	0/20 (0)	7/20 (35)	3/20 (15)	0/20 (0)
Milking parlor										
Teat wipes towels	5/20 (25)	5/20 (25)	2/20 (10)	0/20 (0)	5/20 (25)	2/20 (10)	0/20 (0)	5/20 (25)	2/20 (10)	0/20 (0)
Floor	4/20 (20)	4/20 (20)	0/20 (0)	0/20 (0)	4/20 (20)	0/20 (0)	0/20 (0)	4/20 (20)	0/20 (0)	0/20 (0)
Milking unit	4/20 (20)	4/20 (20)	0/20 (0)	0/20 (0)	4/20 (20)	0/20 (0)	0/20 (0)	4/20 (20)	0/20 (0)	0/20 (0)
Feed bulk truck										
Feed Conveyor	2/20 (10)	2/20 (10)	0/20 (0)	0/20 (0)	2/20 (10)	0/20 (0)	0/20 (0)	2/20 (10)	0/20 (0)	0/20 (0)
Feed tank	1/20 (5)	1/20 (5)	0/20 (0)	0/20 (0)	1/20 (5)	0/20 (0)	0/20 (0)	1/20 (5)	0/20 (0)	0/20 (0)
Wheel	4/20 (20)	4/20 (20)	0/20 (0)	0/20 (0)	4/20 (20)	0/20 (0)	0/20 (0)	4/20 (20)	0/20 (0)	0/20 (0)
Beef slaughterhouse										
Butcher table	3/20 (15)	3/20 (15)	0/20 (0)	0/20 (0)	3/20 (15)	0/20 (0)	0/20 (0)	3/20 (15)	0/20 (0)	0/20 (0)
Knife	1/20 (5)	1/20 (5)	1/20 (5)	0/20 (0)	1/20 (5)	1/20 (5)	0/20 (0)	1/20 (5)	1/20 (5)	0/20 (0)
Floor	4/20 (20)	4/20 (20)	0/20 (0)	0/20 (0)	4/20 (20)	0/20 (0)	0/20 (0)	3/20 (15)	0/20 (0)	1/20 (5)
Poultry slaughterhouse										
Chiller water	3/10 (30)	3/10 (30)	1/10 (10)	0/10 (0)	3/10 (30)	1/10 (10)	0/10 (0)	3/10 (30)	1/10 (10)	0/10 (0)
Floor	4/10 (40)	4/10 (40)	0/10 (0)	0/10 (0)	4/10 (40)	0/10 (0)	0/10 (0)	4/10 (40)	0/10 (0)	0/10 (0)
Conveyor Belt	4/10 (40)	4/10 (40)	1/10 (10)	0/10 (0)	4/10 (40)	1/10 (10)	0/10 (0)	4/10 (40)	1/10 (10)	0/10 (0)
Cutting board	2/10 (20)	2/10 (20)	1/10 (10)	0/10 (0)	2/10 (20)	1/10 (10)	0/10 (0)	2/10 (20)	1/10 (10)	0/10 (0)
Knife	3/10 (30)	3/10 (30)	0/10 (0)	0/10 (0)	3/10 (30)	0/10 (0)	0/10 (0)	3/10 (30)	0/10 (0)	0/10 (0)
Overall (n = 350)	91/350 (26)	91/350 (26)	11/350 (3.1)	0/350 (0)	91/350 (26)	11/350 (3.1)	0/350 (0)	86/350 (24.6)	11/350 (3.1)	5/350 (1.4)

LAMP-LFD, loop-mediated isothermal amplification combined with lateral flow dipstick; PCR, polymerase chain reaction.

1) Culture method was regarded as reference method.

2) TP, true positive: samples detected as positive using LAMP-LFD or LAMP-AGE or PCR were also positive using the reference method; FP, false positive: samples detected as positive using LAMP-LFD or LAMP-AGE or PCR, but negative by the reference method; FN, false negative: samples detected as negative using LAMP-LFD or LAMP-AGE or PCR, but positive by the reference method.

**Table 4:** Kappa values and accuracy of agreement between alternative methods and culture method in animal products (n = 120) and animal production environmental samples (n = 350)

Number of sample	LAMP-LFD	LAMP-AGE	PCR
SE^1)^ (%)	100	100	95.0
SP^2)^ (%)	95.7	95.7	95.7
PPV^3)^ (%)	86.3	86.3	85.7
AC^4)^ (%)	96.6	96.6	95.5
Kappa values^5)^	0.905	0.905	0.878
Total time of detection (h)	1.30	2	2.30

LAMP-LFD, loop-mediated isothermal amplification combined with lateral flow dipstick; PCR, polymerase chain reaction; TP, true positive; TN, true negative; FP, false positive; FN, false negative.

1) SE, sensitivity [TP/(TP+FN)]×100.

2) SP, specificity [TN/(TN+FP)]×100.

3) PPV, positive predictive value [TP/(TP+FP)]×100.

4) AC, accuracy [(TP+TN)/total number of samples]×100.

5) Kappa values [2(TP×TN)–(FP×FN)]/[(TP+FN)×(FN+TN)]+[(TP+FP)×(FP+TN)].
